# HDAC6 depletion improves cystic fibrosis mouse airway responses to bacterial challenge

**DOI:** 10.1038/s41598-019-46555-4

**Published:** 2019-07-16

**Authors:** Julie Rosenjack, Craig A. Hodges, Rebecca J. Darrah, Thomas J. Kelley

**Affiliations:** Departments of Pediatrics, Case Western Reserve University Cleveland, Ohio, USA

**Keywords:** Physiology, Cystic fibrosis, Physiology, Cystic fibrosis

## Abstract

The hypothesis of this study was that Hdac6 depletion would restore cystic fibrosis (CF) responses to bacterial challenge to more wild type profiles using a CF mouse model. CF mice harboring the F508del Cftr mutation respond to bacterial challenge with 25,000 CFU *Pseudomonas aeruginosa* embedded into agarose beads to slow clearance. CF mice respond significantly more aggressively to this challenge compared to WT mice with respect to bacterial clearance, weight loss, neutrophil recruitment, and MIP-2 production. Depletion of Hdac6 expression in the CF mice (CF/Hdac6) significantly improves these responses to more WT levels. Weight loss in response to infection is most severe in CF mice and significantly attenuated in CF/Hdac6 mice. Bacterial levels are reduced at a faster rate in CF/Hdac6 mice compared to CF mice where infection persists. Percent neutrophils in lung lavage fluid post-infection are significantly higher in CF mice, but returned to WT levels with CF/Hdac6 mice. Similarly, CF Mip-2 levels are restored to WT levels in the absence of Hdac6 expression. These data demonstrate that Hdac6 depletion restores CF responses to bacterial challenge to WT-like profiles and offer a potential therapeutic avenue for addressing inflammation and infection in CF airways independently of Cftr correction.

## Introduction

Understanding the CF inflammatory response is an important goal in CF research despite recent advances and success of CFTR-targeted therapies. There are conflicting reports regarding the impact of newer therapies on airway inflammation with an early study stating that no impact on inflammatory markers was observed after six months of treatment despite significant improvement in sweat chloride and lung function measures^1^. However, other studies do indicate a reduction in inflammatory markers though inflammation is still present^2,3^. Though there is not a clear consensus yet regarding the efficacy of CFTR-targeted therapies in dampening inflammation in CF airways, it is likely that complementary anti-inflammatory treatments used in conjunction with CFTR correctors and potentiators would be beneficial. More importantly, patients that harbor CFTR mutations that are not currently responsive to modulators would benefit from therapies that could restore inflammatory regulation.

The ideal anti-inflammatory intervention in CF would restore normal regulation of responses. Restoring proper responses to inflammatory stimuli would still allow for a protective immune response but have a lower risk of dangerous immune suppression. Key to this approach is identifying key regulatory changes in CF that control a number of CF phenotypes that can be therapeutically targeted. We have recently identified changes to microtubule regulation in CF cells that could represent such a therapeutic target^4,5^.

Our previous work has identified two primary alterations to microtubule regulation associated with CF^4,6^. The first of these alterations is slowed microtubule reformation rates after depolymerization in CF primary human nasal epithelial (HNE) cells compared to control non-CF HNE cells^5,6^. This phenotype can be restored with adenosine monophosphate activated protein kinase (AMPK) activators and we have shown that restoration of microtubule formation by ibuprofen may be part of the mechanism of its efficacy^6^. CF microtubules also exhibit less acetylation, a modification that can aid microtubule motor binding and trafficking efficiency^4^. We have demonstrated impaired intracellular trafficking in CF epithelial cells characterized by perinuclear accumulation of free cholesterol in late endosomes/lysosomes consistent with impaired microtubule function^7–10^.

To address the endosomal trafficking impairment in CF cells, we found that trafficking can be restored by increasing tubulin acetylation through inhibition of histone deacetylase 6 (HDAC6), a cytosolic deacetylase that targets tubulin^4^. In cellular studies, we also demonstrated that HDAC6 inhibition reduced inflammatory signaling^4^. These findings were confirmed by a study by Bodas *et al*. and they extended the work to examine the pan HDAC inhibitor suberoylanilide hydroxamic acid (SAHA) in a CF mouse model where inflammation was stimulated by *Pseudomonas aeruginosa* lipopolysaccharide (Pa-LPS) exposure^11^.

We have also demonstrated the broader *in vivo* benefits of HDAC6 inhibition using a CF mouse model carrying the F508del mutation that was crossed to Hdac6 null mice to form a CF mouse lacking Hdac6 expression (CF/Hdac6)^12,13^. CF mice, as well as other animal models, display specific growth defects that correlate to human phenotypes including reduced linear growth and an inability to store fat and gain weight^13–18^. Depletion of Hdac6 expression in our CF mouse model resulted in significantly improved linear growth, increase fat content, and normalized weight gain^12^. Restored growth was also associated with increased IGF-1 levels, a mechanism previously shown to influence growth in CF animal models^12^. Hdac6 knock out is the first intervention to our knowledge not directly targeting CFTR expression that restores growth, particularly linear growth, in a CF animal model. Another phenotype associated with CF animal models is female infertility due to altered hormonal regulation^19^. CF/Hdac6 mice also restored female mouse fertility suggesting broader impacts on CF cell biology^12^.

In this study, we examine the effect of Hdac6 depletion on CF inflammatory responses to a clinical PA isolated using the agar bead model of infection^20,21^. Though not a model of chronic lung infection, this model does recapitulate the CF phenotype of an exaggerated inflammatory response, particularly increased neutrophil recruitment^20–22^. Our data demonstrate that CF/Hdac6 mice have responses to PA-agarose bead challenge that are more representative of WT mice than of CF mice. These data coupled with growth regulation in the CF/Hdac6 mouse demonstrate that microtubule regulation, and HDAC6 signaling in particular, may be a central mechanism in regulating many of the secondary phenotypes associated with impaired CFTR function.

## Results

Previous studies have demonstrated that inhibition of HDAC6 function in CF cells restores microtubule acetylation, intracellular trafficking, and reduces inflammatory signaling^4^. We have also shown that depletion of Hdac6 from CF mice restores growth regulation and normal fat deposition concurrent with increased IGF-1 levels^12^. In this study, we investigated the effects of infection with a clinical isolate of *Pseudomonas aeruginosa* in the *CF/Hdac6* mouse model.

### Depletion of HDAC6 limits weight loss in infected CF mice

**Figure 1:**
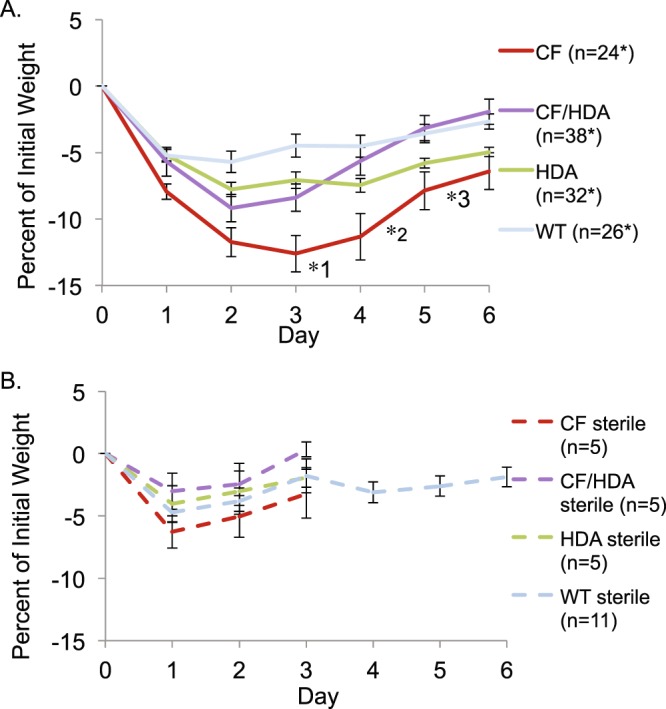
Loss of HDAC6 limits weight decrease in CF/Hdac6 mice. CF, CF/Hdac6 (CF/HDA), *Hdac6* −/− (HDA), and WT mice were weighed to obtain an initial weight and then infected. Mice were weighed daily for 6 days. The percentage of weight loss was calculated based on initial weight and reported. Infected CF mice experienced greater weight loss compared to the other 3 groups throughout the 6-day time course. (**A**) At day 4, CF mice had a significantly greater weight loss of 11.3%, compared to a 5.7% weight loss in CF/HDA mice (*1p = 0.01). CF mice maintained significantly greater weight loss at day 5 compared to CF/HDA mice (7.9% and 3.2% respectively, *2p = 0.01). By day 6, CF/HDA mice recovered most of the initial weight lost (1.94%) compared to CF mice (6.4%, *3p = 0.01). Significance determine by ANOVA with Tukey_Kramer post-hoc test to compare groups. (**B**) Weight loss in response to infection with sterile beads as a control to determine how weight is impacted by bacterial infection and the surgical procedure. for WT, CF, CF/HDA, and HDA mice. Only days 1, 2, and 3 post-infection were tested for CF, CF/HAD, and HAD mice as no weight loss response was observed. WT was carried out to 6 days to examine any longer term effects of surgery. No significant difference in weight loss in response to sterile beads was observed between any groups.

Using this infection model, CF mice exhibit more significant weight loss and slower recovery times compared to WT mice after exposure to bacteria^21^. Weight loss serves as an effective marker as to how mice are able to respond to PAM57 exposure. Infected CF and CF/Hdac6 mice both experienced similar weight loss at days 1, 2, and 3 (Fig. 1A) compared to WT mice. However, by day 4 CF/Hdac6 mice began to recover their weight while CF mice are still affected by the infection. At day 4, CF mice have lost 11.3% of body weight compared to a 5.7% in CF/Hdac6 mice (*1p = 0.01). Interestingly, CF/Hdac6 mice had significantly greater weight loss for days 2 and 3 post-infection compared to infected WT mice (day 2: 9.19% v. 5.71%, p = 0.03; day 3: 8.40% v. 4.48%, p = 0.03), but recover quickly and have no significant differences between WT mice for days 4 to 6. These data suggest that CF/Hdac6 mice have an initial CF-like response to bacterial challenge, but are able to recover more quickly. There was no significant difference between CF/Hdac6 mice and control *Hdac6* −/− mice from days 1–5. At day 6, CF/Hdac6 mice had recovered nearly all of initial percent weight lost compared to control *Hdac6* −/− mice (1.94% vs. 4.97%, p = 0.05). No significant differences were observed between control groups *Hdac6* −/− and WT at any time point (Fig. 1A). As controls, all groups were challenged with sterile agarose beads to determine effects of the procedure on weight loss. No significant differences were observed between any of the sterile bead treated group at any time point (Fig. 1B). Sterile bead treated mice showed no significant weight loss by day 3 so longer term studies were not carried out in most groups. These data demonstrate that depletion of Hdac6 from CF mice leads to a faster and more complete weight recovery compared to CF mice despite an initial weight loss. A summary of statistical comparisons for weight loss in infected mice is shown in Table 1.

**Table 1:** Comparison of weight loss P values between *P*. *aeruginosa* infected mice at various time points.

	Day 1	Day 2	Day 3	Day 4	Day 5	Day 6
CF v. CF/HDA	0.49	0.46	0.07	0.01*	0.01*	0.01*
CF v. HDA	0.01*	0.02*	0.002*	0.19	0.60	0.82
CF v. WT	0.04*	<0.001*	<0.001*	0.007*	0.06	0.10
CF/HDA v. HDA	0.27	0.41	0.53	0.61	0.14	0.05*
CF/HDA v. WT	0.46	0.03*	0.03*	0.93	0.99	0.93
HDA v. WT	0.99	0.59	0.59	0.34	0.40	0.33

Comparisons done by ANOVA with Tukey-Kramer post-hoc test. *indicates significance of individual comparisons.

### Depletion of Hdac6 from CF mice lessens bacterial load

**Figure 2:**
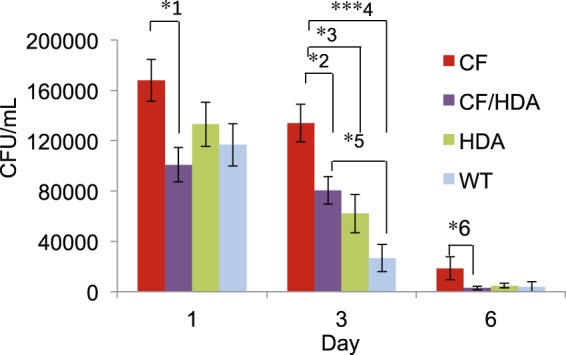
Loss of HDAC6 decreases *P*. *aeruginosa* CFU in infected CF/Hdac6 mice. CF, CF/Hdac6 (CF/HDA), *Hdac6* −/− (HDA), and WT mice were challenged with *P. aeruginosa* clinical isolate PM57. Lung and BAL fluid were harvested at days 1, 3, and 6 and cultured on LB plates at 37 °C overnight. CFUs were counted and CFU/mL were calculated for lung and BAL fluid and added to determine total CFU/mL. CF/HDA mice had significantly less CFU growth compared to CF mice at all 3 time points. At day 1, total CFU/mL from CF mice was significantly greater compared to CF/HDA mice (*1p = 0.03). No significant difference occurred between any of the other groups of mice at day 1. By day 3, CF mice had significantly more CFU growth compared to CF/HDA (*2p = 0.04), HDA (*3p = 0.02), and WT (*4p < 0.0001). A significant difference in CFU growth also occurred between CF/HDA and WT controls at day 3 (*5p = 0.04). By day 6, CFU growth remained lower in CF/HDA mice compared to CF (*6p = 0.04). No significant difference occurred between any of the other groups of mice at day 6. Replicates for days 1, 3 and 6 respectively: CF = 10, 16, 14; CF/HDA = 10, 11, 18; HDA = 10,13, 16; WT = 9, 16, 11. Sterile bead controls had zero CFU growth for all 4 groups of mice (not shown). Significance determine by ANOVA with Tukey-Kramer post-hoc test to compare groups.

In this model, WT mice reduce bacterial load significantly faster that CF mice indicating impaired bacterial killing/clearance in murine CF airways (Fig. 2). To address the rate of bacterial clearance in mice, the amount of total bacteria (CFU/mL) recovered from both bronchoalveolar lavage (BAL) fluid and lung homogenate at different time points post infection was assessed. Infected CF/Hdac6 mice had significantly less recovered bacteria compared to infected CF mice for all 3 time-points (Fig. 2). At day 1, total recovered bacteria from CF/Hdac6 was ~101,000 CFU/mL compared to ~170,000 CFU/mL for CF mice (*1p = 0.03). No significant difference occurred between any of the other groups of mice at day 1. By day 3, CF mice had significantly more CFU growth (~134,000 CFU/mL) than all other groups (CF/Hdac6: ~80,000 CFU/mL, *2p = 0.04; Hdac6 −/−: ~62,000 CFU/mL, *3p = 0.02; WT: 27,000 CFU/mL, *4p < 0.0001). By day 6, bacterial growth remained lower in CF/Hdac6 mice (3000 CFU/mL) compared to CF (19,000 CFU/mL, *6p = 0.04). No significant difference occurred between any of the other groups of mice at day 6. Sterile bead controls had zero CFU growth for all 4 groups. These data demonstrate that loss of Hdac6 on a CF background increases the rate of bacterial clearance or killing and may represent a key regulatory step in CF immune responses. A summary of statistical comparisons of CFU count in infected mice is shown in Table 2.

**Table 2:** Comparison of CFU count (CFU/ml) p-values between *P*. *aeruginosa* infected mice. Comparisons done by ANOVA with Tukey-Kramer post-hoc test. *indicates significance of individual comparisons.

	Day 1	Day 3	Day 6
CF v. CF/HDA	0.03*	0.04*	0.04*
CF v. HDA	0.42	0.002*	0.13
CF v. WT	0.14	<0.001*	0.13
CF/HDA v. HDA	0.52	0.81	0.99
CF/HDA v. WT	0.91	0.04*	0.99
HDA v. WT	0.90	0.25	0.99

### Hdac6 regulates neutrophil recruitment in CF mice

**Figure 3:**
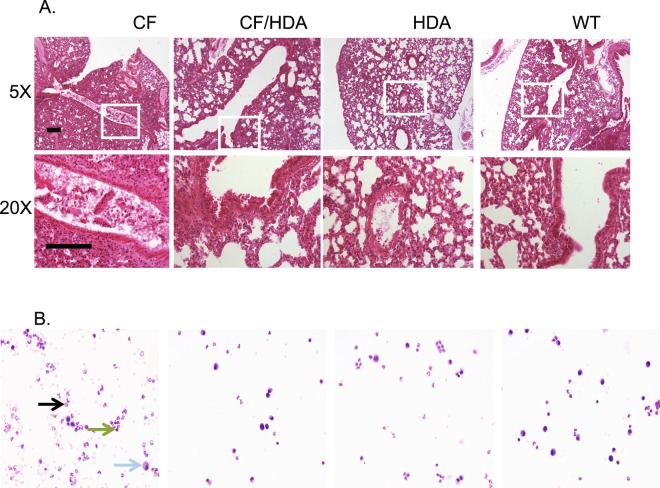
Lung inflammation in *Pseudomonas aeruginosa* (PA) infected mice. (**A**) Representative images of Hemotoxilyn and eosin stained lung sections from F508del (CF), F508del/Hdac6 (CF/HDA), Hdac6 −/− (HDA), and WT mice infected with PAM57-15 PA at day 3 post-infection to show levels of inflammation. Images are shown at 5X and 20X magnification. White box in 5X image highlights area shown in 20X image. (**B**) Representative images of Wright-Giemsa stain of BAL fluid harvested at day 3 shows a denser infiltrate of white blood cells and a higher proportion of neutrophils to monocytes in CF, CF/HDA, HDA, and WT infected mice. The black arrow points to example of a neutrophil and the blue arrow points to a macrophage. Black scale bars represent 140 µm.

**Figure 4:**
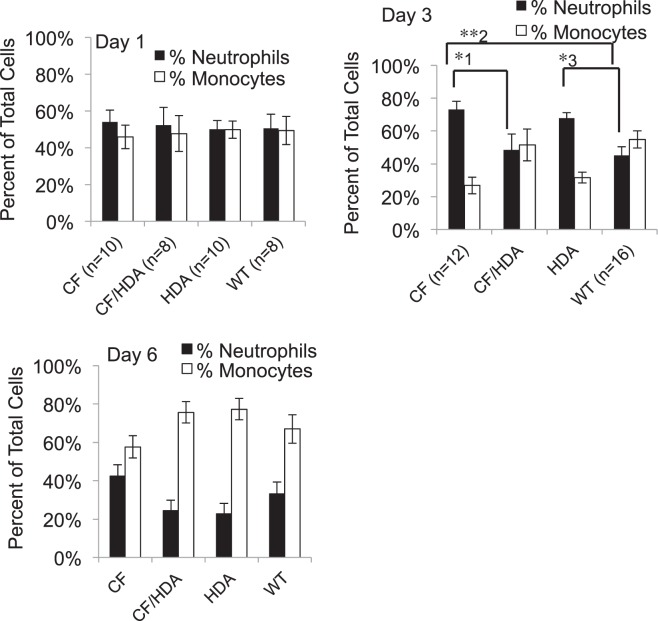
Loss of HDAC6 in infected CF/Hdac6 mice reduces neutrophil recruitment to the airways. CF, CF/Hdac6 (CF/HDA), *Hdac6* −/− (HDA), and WT mice were challenged with *P. aeruginosa* clinical isolate PM57 and analyzed at day 1, day 3, and day 6 post-infection. At day 3, CF mice had a significantly greater percent of neutrophils (73%) to monocytes (27%) compared to CF/HDA (48% and 52%, *1p = 0.04) or WT (45% and 55%, *2p = 0.005). HDA mice also had a significantly greater percent of neutrophils to monocytes compared to WT mice (68% and 32%, *3p = 0.02). Significance determine by ANOVA with Tukey-Kramer post-hoc test to compare groups.

Since recovery and bacterial clearance are improved with the loss of Hdac6 expression in CF mice, the next goal was to examine the inflammatory response. Infected CF/Hdac6 mice exhibit a cellular profile similar to infected WT mice. CF, CF/Hdac6, Hdac6 −/−, and WT mice were infected with 25,000 CFU PAM57-15 as described. Figure 3 shows representative images of hematoxylin stained lung sections at 5X or 20X magnification demonstrating severe inflammatory response in CF mice and reduced infiltrate in subsequent groups (A) and Wright-Giemsa stain of BAL fluid harvested at day 3 which shows a denser infiltrate of white blood cells and a higher proportion of neutrophils to monocytes in CF infected mice compared to CF/Hdac6, Hdac6 −/−, and WT infected mice (B). Lymphocytes constitute less that 0.1% of identified cells at these time points. These data are quantified in Fig. 4. At days 1 and 6, no significant difference occurred between the 4 groups when examining percent neutrophil counts from BAL fluid. Sterile bead challenged mice showed no significant difference in cellular profiles occurred between groups for any of the time points (not shown). At day 3, CF mice had a significantly greater percent of neutrophils (73%) to monocytes (27%) compared to CF/Hdac6 (48% and 52%, *1p = 0.04) or WT (45% and 55%, *2p = 0.005) (Fig. 4). *Hdac6* −/− mice also had a significantly greater percent of neutrophils to monocytes compared to WT mice (68% and 32%, *3p = 0.02) but not compared to either CF or CF/Hdac6 mice (Fig. 4). Whether reduced neutrophil counts are due to direct effects of Hdac6 depletion on neutrophils or due to reduced recruitment signaling is unclear, but a more WT inflammatory cell profile clearly results from Hdac6 depletion in CF mice. By day 6, the neutrophil response had resolved in all mouse models. Percent neutrophils were still elevated in the CF mouse mice (41%), but not statistically different that other models. Though not statistically different, the data indicate that Hdac6 depletion from CF mice helps resolve the inflammatory response in a more WT-like manner. These data are consistent with bacterial clearance rates shown in Fig. 2 suggesting that perhaps enhanced clearance of bacteria may be key to resolving inflammation. A summary of statistical comparisons of percent neutrophils in infected mice is shown in Table 3.

**Table 3:** Comparison of percent neutrophils p-values between *P*. *aeruginosa* infected mice. Comparisons done by ANOVA with Tukey-Kramer post-hoc test. *indicates significance of individual comparisons.

	Day 1	Day 3	Day 6
CF v. CF/HDA	0.99	0.04*	0.16
CF v. HDA	0.98	0.93	0.12
CF v. WT	0.98	0.005*	0.52
CF/HDA v. HDA	1.00	0.12	0.99
CF/HDA v. WT	1.00	0.98	0.99
HDA v. WT	0.99	0.02*	0.96

### Cytokine production is reduced in Hdac6-depleted CF mice

**Figure 5:**
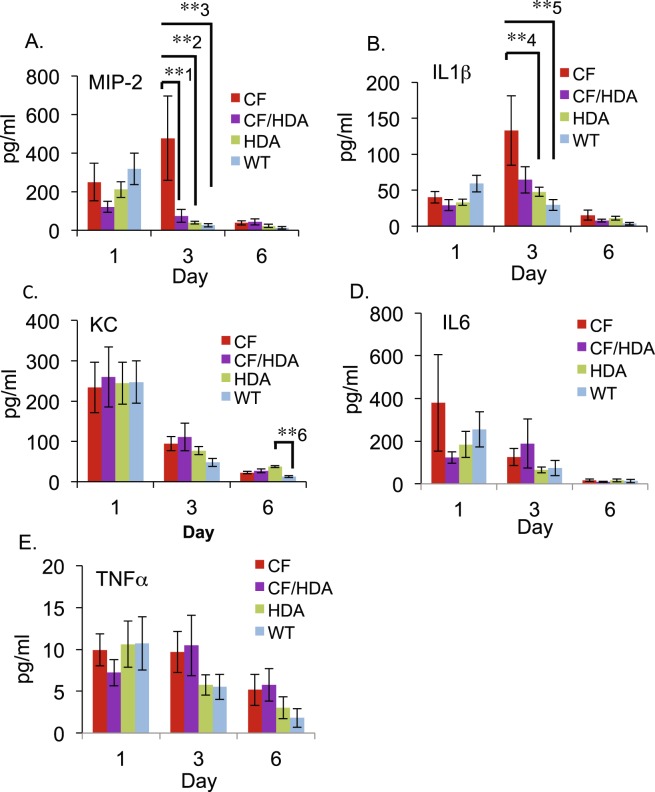
Effect of Hdac6 depletion on cytokine production. CF, CF/Hdac6 (CF/HDA), *Hdac6* −/− (HDA), and WT mice were challenged with *P. aeruginosa* clinical isolate PM57. Cytokine production at days 1, 3, and 6 post-infection for: (**A**) Mip-2. CF mice had significantly higher levels at day 3 compared to CF/HDA (**1p = 0.006), HDA (**2p = 0.002), and WT (**3p = 0.001). (**B**) IL-1β. CF mice had significantly higher levels of IL-1β at day 3 compared to both HDA (*4p = 0.03) and WT (**5p = 0.006). (**C**) KC. At day 6, HDA mice had significantly higher levels of KC compared to WT (**6p = 0.007). (**D**,**E**). No significant differences appeared for inflammatory cytokines IL-6 or TNF-α between any of the groups at the given time points. Replicates for days 1, 3,and 6 respectively: CF = 10, 6, 12; CF/HDA = 9, 9, 16; HDA = 9, 11, 16; WT = 9, 12, 11. Significance determine by ANOVA with Tukey-Kramer post-hoc test to compare groups.

In order to address whether inflammatory signaling may be impacted by Hdac6 depletion, cytokine levels were examined. Expression levels of IL-1β, Mip-2, KC, TNF-α, and IL-6 were measured. IL-1β was measured to determine the production of pro-inflammatory cytokines that have paracrine effects of promoting further inflammatory responses. KC, Mip-2, and IL-6 were measured as an assessment of production of NF-κB-dependent genes. Mip-2 expression was significantly elevated in BAL fluid from infected CF mice at day 3 post-infection, while levels of Mip-2 in infected CF/Hdac6 mice are similar to control groups *Hdac6* −/− and WT (CF: 476 pg/mL, CF/Hdac6: 75 pg/mL, (**1p = 0.006), Hdac6 −/−: 39 pg/mL (**2p = 0.002), and WT: 26 pg/mL, (**3p = 0.001)) (Fig. 5A). Infected CF mice also had significantly higher levels of IL-1β at day 3 (133 pg/mL) compared to both *Hdac6* −/− (48 pg/ml, *4p = 0.03) and WT (29 pg/mL, **5p = 0.006) (Fig. 5B). Although lower in CF/Hdac6 mice, the reduction does not meet statistical significance. At day 6, *Hdac6* −/− mice showed higher levels of KC (37 pg/mL) compared to WT (12 pg/mL, **6p = 0.007) (Fig. 5C). No significant differences appeared for inflammatory cytokines IL-6 (Fig. 5D.), and TNF-α (Fig. 5E) between any of the groups at the given time points. Sterile bead challenged controls showed no significant differences between any of the groups for any of the given time points (not shown).

Cytokine responses are comparable to previous studies using this model. The original study by van Heeckeren *et al*. showed that Pseudomonas challenge of CF mice resulted in significant increases in TNF-α, mip-2, and KC levels in CF mice compared to control WT mice^23^. The cytokine profile we observe is slightly different with IL-1β and mip-2 levels being significantly elevated in CF. One significant difference between our study and the original is the dose of *Pseudomonas aeruginosa*. The original study inoculated with approximately 1.0 × 10^5^ CFU, where this study used 2.5 × 10^4^ CFU for the challenge to reduce mortality. The lower inoculum in our study produces a less intense cytokine response and likely accounts for more variability in the response.

## Discussion

There is still a lack of understanding as to how the loss of CFTR function leads to many of the secondary manifestations of CF disease. We have previously identified that microtubule regulation is altered in CF cells and tissues resulting in impaired intracellular transport, particularly of endosomes, which can be visualized by cholesterol staining^4–10,24^. Microtubule changes related to CF include reduced tubulin acetylation and reduced rates of tubulin re-polymerization^4–6^. Our previous studies have shown that inhibition of HDAC6 function restores intracellular transport in CF cells and reduced NF-κB activation suggesting a relationship between microtubule regulation and CF inflammatory responses^4^. This relationship is supported by a recent study by Bodas *et al*. that demonstrated that pan-HDAC inhibitors and an HDAC6 inhibitor reduced inflammation in CF mice challenged with lipopolysaccharide^11^. However, the study by Bodas *et al*. utilized an i.p. injection of Pa-LPS to stimulate a systemic inflammatory response and then measured lung cytokines. Although likely a valid test of the role of HDAC inhibitors in controlling inflammation, the approach is not an approximation of CF airway response to bacterial challenge. We utilize a well-tested model of clinical PA isolates embedded in agarose beads to slow clearance placed directly into the lung to examine lung-specific responses to bacterial infection^20,21,23,25–27^. Though not a model of chronic infection, this technique has been shown to model aggressive CF inflammatory responses in mice and allows for the assessment of potential anti-inflammatory interventions. Secondly, we utilize a genetic ablation of Hdac6 expression as opposed to pharmacological inhibition. This approach eliminates the possible contributions of off-target drug effects and specifically examines the role of Hdac6 in CF inflammation control.

The goal in developing a new an anti-inflammatory therapy for CF is to find an intervention that maintains an immune response so infections can be eradicated, but to also allow for normal resolution of the inflammatory response so prolonged, aggressive inflammation observed in CF airways is dampened. Consistent with previous cell studies showing benefit of HDAC6 inhibition on CF cellular phenotypes, here it is shown that *in vivo* depletion of Hdac6 in a CF mouse model largely reverses CF responses to bacterial challenge to a more WT profile. In the broad measure of weight loss and recovery, CF/Hdac6 mice lose weight initially at a rate consistent with CF mice, but quickly recover and restore the weight to that seen in WT mice. These data suggest the ability of CF/Hdac6 mice to mount an immune response, but to quickly resolve both the infection and inflammation. More telling, the PA-agarose bead model does recapitulate the neutrophil-dominant immune response in CF mice characteristic of human CF airway disease^22^. CF/Hdac6 mice exhibit an inflammatory profile more consistent with WT mice that CF mice, again suggesting an appropriately mounted immune response and resolution. Another caveat to this approach is that it has been reported that HDAC6 inhibition can lead to diminished immune response due to impaired macrophage recruitment^28^. Our data do not show a significant difference in macrophage numbers but we do observe a modest increase in neutrophil counts in *Hdac6* −/− compared to WT mice. The effects of HDAC6 inhibition on broader immune functions needs to be assessed, but our data demonstrate that Hdac6-depletion reverses CF inflammatory phenotypes.

Despite the success of Hdac6 depletion on the CF response to bacterial challenge, areas of caution remain that will need further investigation. Previous studies have implicated a role for defective autophagy as a mechanism leading to excessive inflammatory responses in CF^29–35^. HDAC6 function is a key component of the autophagy machinery and inhibition of HDAC6 could further deplete the autophagic response and lead to poor bacterial clearance and more inflammation^36–40^. However, there is also significant evidence that HDAC6 inhibition reduces inflammation in various conditions and model systems^11,41–44^. Our data and those of Bodas *et al*. clearly show a benefit of HDAC6 inhibition in a CF context^11^. Since autophagy is already defective in CF cells, the adverse effects of HDAC6 inhibition on autophagic processes may be invisible in a CF context and the anti-inflammatory benefits are easier to observe. The relationship between HDAC6 and autophagy in CF will need to be further explored to fully evaluate the risks and benefits of HDAC6 inhibition as a potential CF therapy, particularly when considering long-term effects.

Mechanistically, the reduction of Mip-2 expression is consistent with dampened NF-κB activation upon Hdac6 depletion as our previous cellular studies show^4^. However, the cytokine response is dampened since we use a lower inoculum of bacteria. We reduced the inoculum compared to other studies to increase survival while still maintaining the hyper-neutophilic response in the model. One potential mechanism of efficacy is that Hdac6 depletion is improving F508del CFTR function in our mouse model as HDAC6 inhibition has been shown to improve F508del trafficking^45,46^. However, we have shown in our previous work that CFTR function is unaffected by Hdac6 depletion in this mouse model^12^. Using the nasal potential difference assay to assess CFTR function, we observed that depletion of Hdac6 expression had no impact on the F508del CFTR function in mice. In this mouse model, however, the expression of F508del CFTR is very low and is functioning essentially as a knock-out model^47^. Given these findings and the nature of this particular F508del Cftr mouse model, augmentation of CFTR function by Hdac6 depletion is an unlikely and the mechanism for the anti-inflammatory benefits are CFTR-independent.

Another mechanistic consideration to be considered is whether Hdac6 depletion is acting through innate or adaptive immunity mechanisms as HDAC6 function has been shown to influence both systems. Wang *et al*. have shown that HDAC6 inhibition provides resistance to *Mycobacterium tuberculosis* infection and that HDAC6 inhibition is acting through both mechanisms^48^. This manuscript shows that by 14 to 21 days post-infection, the presence of the HDAC6 inhibitor tubistatin A enhances T-cell migration into the lung of infected mice. This finding is consistent with other studies showing enhanced T cell responses in different systems after HDAC6 inhibition^49,50^. Our current study only examines out to 6 days post-infection, so we are not able to make any conclusions regarding the adaptive immune response with our current model. Our data demonstrate that our Pseudomonas isolate is cleared by 10-days post-infection with no treatment (not shown), so longer term studies are not possible at the current inoculation dose. Interestingly, counter to both our study and that by Bodas *et al*., Wang *et al*. find that HDAC6 inhibition leads to increased pro-inflammatory cytokine production that signals an increase in innate immunity. Our study is done in the context of CF, which has an inherently different innate immune response and the studies utilize different infections agents, but the impact of HDAC6 on adaptive immunity needs to be further explored.

The greater significance of this study is seen in combination with our previous study demonstrating that CF/Hdac6 mice demonstrate improved linear growth and weight gain compared to CF mouse controls^12^. Several studies have shown a strong correlation between body mass index (BMI) and severity of lung disease in CF patients^51–55^. It is postulated that either severe catabolism initiated by aggressive lung disease reduces BMI, or conversely, that lower BMI reduces airway capacity leading to more rapid lung disease. Our studies suggest that BMI and airway disease are strongly correlated, because the same HDAC6-dependent pathway regulates both outcomes. The prospect that multiple key CF phenotypes can be addressed by HDAC6 inhibition is important for patients with CFTR mutations not currently responsive to potentiator and corrector therapies, or to even augment CFTR-targeted therapies to improve inflammation and infection management.

In summary, this study demonstrates that Hdac6 depletion from a CF mouse model reverts multiple aspects of the CF inflammatory response to WT profiles. With the improvement in the airway response to bacterial challenge and with the correction of growth phenotypes, HDAC6 represents a potentially potent therapeutic target. HDAC6 inhibitors such as ACY-1215 are currently in clinical studies for the treatment of multiple myeloma and have been found to have anti-inflammatory influences in osteoarthritis studies^42,56^. HDAC6 inhibition could have significant beneficial effect for CF patients independently of CFTR genotype.

## Methods

### Mice

To create mice expressing F508del CFTR and deficient in HDAC6 expression, we crossed mice with the *Cftr* mutation *F508del*^47^ with HDAC6 null mice^57^ as previously described^12^. Both strains were on a C57Bl/6 J background. To decrease the incidence of intestinal obstruction that is common in CF mice, mice were allowed access to sterile water with osmotic laxative, PEG-3350 with electrolytes (Kremers Urban Pharmaceuticals). All mice were maintained on a 12 h light, 12 h dark cycle at a mean ambient temperature of 22 °C. The Institutional Animal Care and Use Committee (IACUC) of Case Western Reserve University approved all animal protocols. All methods were conducted according to necessary guidelines and established regulations.

### Preparation of *P. aeruginosa*-laden agarose beads

*P*. *aeruginosa* labeled with mCherry (mCH PA M57-15) was streaked on a 0.3 mg/ml gentamycin/LB agar plate and incubated at 37 °C overnight. Five colonies of mCH PA M57-15 were collected and added to 25 ml of LB broth and incubated at 37 °C, 200 rpm, for 18–20 hours. After 20 hours, the optical density at 600 nm of the mCH PA M57-15 was measured to obtain an absorbance of ~0.3 for a 10-fold dilution. Bacteria were stored on ice until ready to use.

Two 600-mL beakers containing 250 mL sterile mineral oil and stir bars and a flask of sterile 2% agarose in PBS were warmed in a 50 °C water bath for 30 minutes. The 2 beakers of mineral oil were placed in ~6 × 10 inch plastic container and then placed on magnetic stir plates and set at a medium-high stir to form a vortex. 23 mL of warm, dissolved agarose/PBS was pipetted into one of the beaker of mineral oil to form sterile beads. 5 mL of mCH PAM57-15 was pipetted into the remaining agarose/PBS and mixed and then 23 mL of the mCH PAM57-15/agarose/PBS mixture was pipetted into the other beaker of mineral oil. After 6 minutes of stirring, 20 g of ice was added to the plastic container every minute for 10 minutes while monitoring the stirring to ensure a constant vortex. After 10 minutes, the bead/oil mixture was poured into 50 mL conical tubes containing 15 mL of pre-warmed 0.5% SDC/PBS and spun at 4 °C, 3000 g, for 15 minutes to separate the beads on the bottom from the top layer of oil. The oil was removed and the top half layer of beads were pipetted into 50 ml conical tubes containing 20 mL of pre-warmed 0.25% SDC/PBS and spun under same conditions. After the second wash, any oil/PBS was removed to ~15 mL and the beads were pipetted into 50 ml conical tubes containing 20 mL of PBS and washed 4×. After the final wash, the PBS was removed to a 3:4 volume of beads to PBS. A 100ul aliquot of the bead mixture was used to image and measure the size of beads, which should be ~20–600 µm. The beads were viewed under the red channel to ensure sterile beads are sterile and bacterial-laden beads have mCH PA M57-15. A 1:50 dilution of the beads was homogenized on highest speed for 1 minutes and serial diluted 10-fold 10 times. The dilutions were plated on a 0.3 mg/mL gentamycin/LB agar plate and incubated at 37 °C overnight. The following day, the CFU/mL were calculated the bead/PBS mixture was diluted to a concentration of 25,000 CFU/50 µL.

### Inoculation of mouse airway with agarose beads

All animal studies were approved by the CWRU IACUC committee, and all experiments were performed in accordance with relevant guidelines and regulations. The mice receiving sterile bead treatment were used first to avoid cross contamination. The mouse was anesthetized with isoflurane via inhalation with a nosecone. Ophthalmic ointment was applied the eyes and the thorax was sterilized with isopropanol. A ~1 cm midline median incision was made using surgical scissors in the tracheal region. The tracheal muscles were gently teased apart to expose the trachea and a ~1 mm transverse incision was made between the tracheal cartilage. A 1 mL syringe fitted with a 22G × 1″ flexible catheter was used to mix and draw up the bead/PBS mixture leaving no dead space in catheter. The catheter/syringe was immediately inserted into the tracheal incision and 50 mL of the bead/PBS mixture was injected into the lungs of the mouse to administer 25,000 CFU per mouse. The mouse was placed into a clean, sterile cage on top of a warming pad and monitored until out of anesthesia. The weight and condition of inoculated mice were monitored until the day of harvest.

### Harvest

Sterile bead inoculated mice were harvest first to avoid cross contamination. The mouse was euthanized by CO_2_ inhalation, placed in the supine position, and disinfected with 70% ethanol. A midline, median incision was made to expose the thoracic cage and the diaphragm cut to expose the lungs. A 22G × 1″ flexible catheter was inserted between the tracheal cartilage to just before the bronchi. Surgical thread was tied around trachea and catheter. BAL fluid was collected using a syringe filled with 1 ml PBS inserted into the catheter and injected into and withdrawn from lungs. Ten microliters of BAL fluid was used to determine total white blood cell count, another 15 µL was plated on 0.3 mg/mL gentamycin/LB agar for CFU/µL count, and 200 µL of BAL fluid was used to determine cell differentials using Giemsa/Miller staining. The BAL fluid was then spun at 4 °C, 500 g, 10 minutes. The supernatant was used for cytokine analysis using Luminex. The lungs were removed and rinsed with PBS and placed in 2 mL tubes containing zirconium homogenization beads and homogenized and 15 µL plated on 0.3 mg/mL gentamycin/LB agar for CFU/µL count. The remaining lung homogenate was spun at 4 °C, 5000 g, 10 minutes and the supernatant removed. The remaining supernatant was stored at −80 °C. For staining of lung tissue, 10 μm sections were fixed in formalin, embedded in paraffin, deparaffinized in xyleneand ethanol, and stained with hematoxylin and eosin by the histology core facility at CWRU.

## Data Availability

The datasets generated during and/or analyzed during the current study are available from the corresponding author on reasonable request.
